# Acute DWI Reductions In Patients After Single Epileptic Seizures - More Common Than Assumed

**DOI:** 10.3389/fneur.2018.00550

**Published:** 2018-07-25

**Authors:** Annemarie Hübers, Katharina Thoma, Michael Schocke, Susanne Fauser, Albert C. Ludolph, Jan Kassubek, Elmar H. Pinkhardt

**Affiliations:** ^1^Department of Neurology, University of Ulm, Ulm, Germany; ^2^Epilepsiezentrum Bethel, Krankenhaus Mara, Bielefeld, Germany

**Keywords:** magnetic resonance imaging, diffusion weighted imaging, epilepsy, seizure, status epilepticus

## Abstract

**Background:** Changes of cerebral diffusivity detected by magnetic resonance imaging (MRI) have been reported in epilepsy. Diffusion weighted imaging (DWI) detects changes in the distribution of water molecules by measuring the apparent diffusion coefficient (ADC) and is mainly used in the diagnosis of ischemic stroke. DWI changes in epilepsy were reported in status epilepticus (SE) or series of seizures. It remains unclear whether this phenomenon also occurs after single seizures. Accordingly, possible pathomechanisms have only been discussed on the presumed basis of ongoing epileptic brain activity.

**Methods:** In this retrospective study, we systematically analyzed DWI alterations related to epileptic seizures in 454 patients who received MRI scanning within the first 24 h after seizure onset.

**Results:** DWI restrictions not classified as ischemic stroke were observed in 18 patients (4%). We found DWI restrictions in 19% of patients with SE/seizure series and in 3% of patients after single focal and 2.5% after single generalized seizures. 17 patients with DWI alterations were diagnosed with a structural epilepsy. DWI signal decreased in the majority of patients within the first days and could not be detected in follow-up imaging >3 months. In all patients except one, DWI alterations were detected in the same hemisphere as the lesion. In the case of seizure series or SE, DWI restrictions mostly presented with a typical “garland-like” pattern alongside the cortical band or on the border of a defined lesion, while in isolated seizures, the restrictions were often rather subtle and small.

**Discussion:** We show that DWI restrictions can be observed in patients after single epileptic seizures. As the vast majority of these patients was diagnosed with an epilepsy due to structural cerebral pathology, DWI restriction may reflect a higher vulnerability in these regions. This might also explain the fact that diffusivity changes were observed after single focal seizures as well as after multiple seizures or SE. The occurence itself on one side as well as the spatial pattern of this phenomenon on the other may thus not only be related to the duration of ictal activity, but to structural pathology.

## Introduction

Epilepsy is a clinical condition characterized by recurrent transient seizures due to abnormal synchronous and/or excessive neuronal activity of cortical neurons. Patients experiencing a seizure may present with motoric, sensoric or cognitive symptoms.

Since it was first described in 1929 by Hans Berger, the electroencephalogram (EEG) has been used as the main diagnostic tool to detect and localize epileptic neuronal activity. In recent years, magnetic resonance imaging (MRI) has become more and more important in the diagnostic workup of epilepsy patients. In clinical routine, imaging techniques are mainly used in order to search for structural cortical abnormalities or lesions representing possible epileptogenic foci. Yet, not only structural, but also functional changes associated with epileptic brain activity can be visualized by neuroimaging. In 1933, Penfield described for the first time hemodynamic changes associated with seizures in an angiographic study (1). Since, hemodynamic alterations in epilepsy patients have been reported in several studies using different techniques such as perfusion MRI (2), blood oxygenation level-dependent (BOLD) MRI (3) and single photon emission tomography (SPECT) (4). As MRI techniques became more advanced in the course of the last few years, apart from hemodynamic changes, diffusion-weighted MRI (DWI) became a new focus of scientific interest. DWI detects changes in the distribution of water molecules in the brain by measuring the apparent diffusion coefficient (ADC). It is a sensitive technique to study the molecular motion of water, i.e. water diffusivity, which represents an intrinsic characteristic of tissues (5). In clinical routine, DWI is mainly used in the diagnosis of acute ischemic stroke, but also brain tumors, cerebral abscesses etc. As for its application in epilepsy, some older and more recent case reports (6–10) and smaller studies report changes in DWI associated with epileptic brain activity. In 2011, Chatzikonstantinou et al. (11) published a prospective series of 54 selected patients with DWI alterations related to status epilepticus. They describe hippocampal DWI abnormalities in the majority of patients and EEG changes that correlated with lateralization of DWI abnormalities. No correlation between DWI changes and clinical features could be observed. In contrast, Rennebaum et al. (12) reported on a series of 19 out of 69 cases with cortical and thalamic DWI restrictions in patients with status epilepticus (SE) who presented more often with quantitative disorder of consciousness compared to patients without DWI abnormalities. The first prospecitve study on patients with SE or seizure series was published by Jabeen et al. in 2017 (13), who observed DWI abnormalities in 20 out of 30 patients. To the best of our knowledge, apart from these studies, no larger series of patients with DWI alterations associated with epileptic brain acitivity have been published so far. Thus, many questions remain unanswered. In particular, DWI alterations have almost exclusively been observed in patients with series of seizures or SE, whereas it remains unclear whether this phenomenon also occurs in patients after single focal or generalized seizures. Accordingly, possible pathomechanisms have only been discussed on the presumed basis of an ongoing epileptic brain activity.

Another question is whether specific patient groups like patients with existing brain lesions as a cause of their epilepsy are more prone to diffusivity changes then patients with other forms of epilepsy.

To adress these questions, we performed a retrospective study on a large cohort of 454 patients with acute epileptic seizures or SE who received an MRI within the first 24 h after seizure onset.

## Materials and methods

### Patient collective

We studied a collective of 454 patients who presented at the Department of Neurology, Ulm University Hospital, Germany, between June 2009 and March 2013 with an acute (i.e., within the last 24 h prior to admission) epileptic seizure (focal, generalized or secondary generalized), a series of seizures (i.e., ≥ 2 seizures within the last 24 h) or SE and who received an MRI scan within the *first* 24 h after or during epileptic symptoms. The study was approved by the ethics committee of the University of Ulm (Application No. 237/15).

### Clinical data

We recorded seizure semiology and type of epilepsy syndrome according to the revised terminology of the International League Against Epilepsy (14). Diagnosis in the individual patient was based on clinical findings, anamnestic information and EEG findings. SE was defined as >5 min of ongoing seizure activity for generalized tonic-clonic seizures (GTCS, GSE) (15–17) and >20 min for focal seizures without impairment of consciousness or awareness (FACS, FACSE) or dyscognitive focal seizures (FICS, FICSE) or as a series with two or more seizures without full clinical recovery in between (17, 18). A series of seizures was defined as two or more seizures in the last 24 h prior to admission with full recovery in between.

### MRI data

MRI was performed on a 1.5 Tesla MRI system (TIM Symphony, Siemens, Germany). With a 5 mm slice thickness, standardized T2 (repetition time (TR) 4,000–6,000 ms, time to echo (TE) 102 ms), T1 (TR 573 ms, TE 12 ms), fluid attenuated inversion recovery (FLAIR, TR 6180 ms, TE 112), T2^*^ (TR 800–1,000 ms, TE 24 ms) and DWI (TR 4,000–6,000 ms, TE 100 ms) were acquired. ADC maps were obtained from DWI. All MRI data sets were obtained in the same orientation and slice positions. We recorded all findings in the first MRI performed after seizure activity, as well as on subsequent MRIs at our department and the time between seizure onset and first MRI. DWI abnormalities were only accepted if a high contrast to background was detectable, or a corresponding reduction of ADC was present and artifacts could be excluded. Furthermore, DWI alterations were only classified as seizure-related when they were cortical or localized in adjacent subcortical areas and not respecting vascular territories or when they were restricted to the hippocampal area or pulvinar thalami.

### EEG recording

EEG was performed with a standard 10–20 system (21 electrodes) over 15 to 30 min using a digital EEG-acquisition and analysing system (Natus, Germany) and evaluated by a board-certified neurologist. Frontal, temporal, parietal and occipital regions were studied in each patient. Artifacts were identified by visual inspection. Localization of abnormal EEG patterns was identified by the electrodes over which the patterns were maximally visible, i.e., highest amplitudes in referential derivations or phase inversion in bipolar derivations (19).

### Statistical analysis

Statistical analyses were carried out using IBM SPSS Statistics21.0 (IBM Corporation, Armonk, New York, USA). We compared the total group of patients with peri-ictal DWI restriction (*n* = 18) with the total group without DWI restriction (*n* = 436) using the Chi-square test (or Fisher's exact test). A statistical significance was assumed if *p* < 0.05.

## Results

In total, we identified 18 out of 454 patients (4.0%) with DWI abnormalities associated with epileptic symptoms (4 males, 14 females, mean age 71.1 years). Six patients in this group (33.3%) presented with a SE (five focal, one generalized) and one patient (5.6%) with a series of focal seizures leading to admission. Five patients were diagnosed with a single GTCS (27.8%), six with single focal seizures (33.3%). As for etiology, 17 patients (94.4%) were diagnosed with structural epilepsy, one patient with epilepsy of unknown etiology. Clinical, EEG and MRI data of the patients with DWI alterations are summarized in Table 1. Twelve patients in this group received a follow-up MRI, eight within the first 7 days after seizure or SE and four within 3–31 months (see Table 1). In the majority of patients, DWI restrictions decreased significantly or dissolved completely during the first days after epileptic activity. None of the patients who received a follow-up after 3 months or later exhibited DWI changes any more.

**Table 1:** Clinical, EEG, and MRI characteristics of patients with DWI reductions after seizure or SE.

**Patient No**.	**Age range**	**Clinically affected hemisphere**	**Etioloqv**	**Semioloqv**	**Presumed cause**	**Structural MRI pathology localization**	**DWI localization**	**DWI localization follow-up MRI**	**EEG pattern**	**EEG pattern spatial characteristics**	**EEG pattern temporal characteristics**
1	76–80	Unknown	Symptomatic	FICS	Post-ischemic gliosis	Right frontal	Right frontal		Normal	N.A.	N.A.
2	90–95	Unknown	Symptomatic	FICS	SVE	Bi-hemispheric	Left temporal/ Lippocampal		Slowing	Generalized	Continuous
3	56–60	Unknown	Symptomatic	GTCS	Glioblastoma	Right frontal	Right frontal		Slowing	Right frontal	Continuous
4	70–75	Unknown	Symptomatic	GTCS	Post-ischemic gliosis	Left Fronto-temporal and parietal	Left parietal		Slowing	Right hemisphere, Left hemisphere	Continuous, Discontinuous
5	66–70	Right	Symptomatic	GTCS	CAA	Bi-hemispheric	Right parietal		Sharp-waves, Slowing	Right temporo-parieto-Occipital, Left hemisphere	Continuous
5	80–85	Left	Symptomatic	FACS	SVE	Bi-hemispheric	Right frontal	Right frontall with decreased signall after 5 days, Completely reversible after 7 weeks	Normal	N.A.	N.A.
7	70-75	Unknown	Symptomatic	GSE	Post-ischemic gliosis	Bi-hemispheric	Left and right frontal, parietal, temporal, occipital	Right frontal, parietal, Occipital, decreased signal after 4 days	Sharp-waves	Right fronto-central	Continuous
8	76–80	Left	Symptomatic	FICS	Post-ischemic gliosis	Left occipital	Left occipital	Completely reversible after 4 months	Slowing	Left temporal	Continuous
9	86–90	Unknown	Symptomatic	FICS	SVE	Bi-hemispheric	Right temporal/ lippocampal	Completely reversible after 1 day	No EEG	N.A.	N.A.
10	36–40	Right	Symptomatic	GTCS	Cerebral cysticercosis	Bi-hemispheric	Right thalamus	Completely reversible after 17 months	Slowing	Right temporal	Continuous
11	70–75	Unknown	Symptomatic	GTCS	Limbic encephalitis	Left temporal and hippocampal	Left temporal/ lippocampal	Left temporal after 7 days, Completely Revesible after 3 Months	Slowing	Generalized	Continuous
12	60–65	Riqht	Symptomatic	FICSE	SVE	Bi-hemispheric	Right parietal, occipital, :halamus		Sharp-waves, Slowing	Right frontal, right hemisphere	Discontinuous, Continuous
13	40–43	Left	Symptomatic	FACSE	Astrocytoma	Left central	Left occipital	Competely reversible after 3 months	Slowing	Bi-frontal	Continuous
14	86–90	Left	Symptomatic	FICS	SVE	Bi-hemispheric	Left and right Frontal	Completely reversible after 3 days	Slowing	Left fronto-temporal	Continuous
15	60–62	left	Symptomatic	FICSE	Post-ischemic gliosis	Bi-hemispheric	Left temporal/ lippocampal, oarietal, :halamic	Completely reversible after 2 days	Sharp-waves	Bi-frontal->left frontal-generalized	Continuous
16	70–75	Both	Unknown	FICS series	Unknown	Left and right occipital	Left and right occipital	Left and right occipital after 7days	Slowing	Left temporo-occipital	Continuous
17	76–80	Unknown	Symptomatic	FICSE	SVE	Bi-hemispheric	Left hippocampal	Left hippocampal, decreased signal after 4 days	Sharp-waves vs. triphasic waves	Bi-frontal	Discontinuous
18	76–80	Unknown	Symptomatic	FICSE	SVE	Bi-hemispheric	Left parietal	Completely reversible after 31 months	Sharp-waves	Left hemisphere	Continuous

From the 436 patients without DWI restriction, the majority was diagnosed with a structural epilepsy (336 patients in total, 77.1%). Yet, this percentage was smaller then in the patient group with DWI restriction, although this difference did not reach statistical significance (*p* = 0.19). Only 25 patients in total were diagnosed with a genetic epilepsy (5.7%), and 44 patients with an epilepsy of unknown origin (10.1%). 20 patients presented with a situation-related seizure due to alcohol withdrawal and seven with a situation-related seizure due to other causes (hypoglycaemia, substance abuse, electrolyte imbalance). Four patients presented with a single occasional convulsion. The percentage of women was higher in the DWI-restriction-group with 14 female vs. only four male patients here, whereas in the non-DWI-restriction group, sex distribution was even (218 male vs. 218 female). Patients with DWI restriction were slightly older (mean age, 64.5 ± 19.9 years in the group without DWI restriction vs. 71.1 ± 14.1 years in the group with DWI restriction).

Two hundred and fifteen patients in total (47.4%) were diagnosed with a single focal seizure, six of which (2.8%) showed a DWI restriction on MRI. From 201 patients who presented with a single generalized seizure, five patients (2.5%) showed DWI abnormalities and from 23 patients with a series of GTCS or GSE, DWI restriction was observed in one patient (4.3%). Nine patients presented with a focal SE, five of them (55.6%) showing alterations in the DWI sequence. That is, from 32 patients with a series of seizures or focal or generalized SE, 18.8% (six patients) showed a DWI restriction.

In six patients without DWI restriction, exact seizure semiology could not be determined retrospectively.

### Relation between structural MRI pathology and DWI restriction

**Figure 1:**
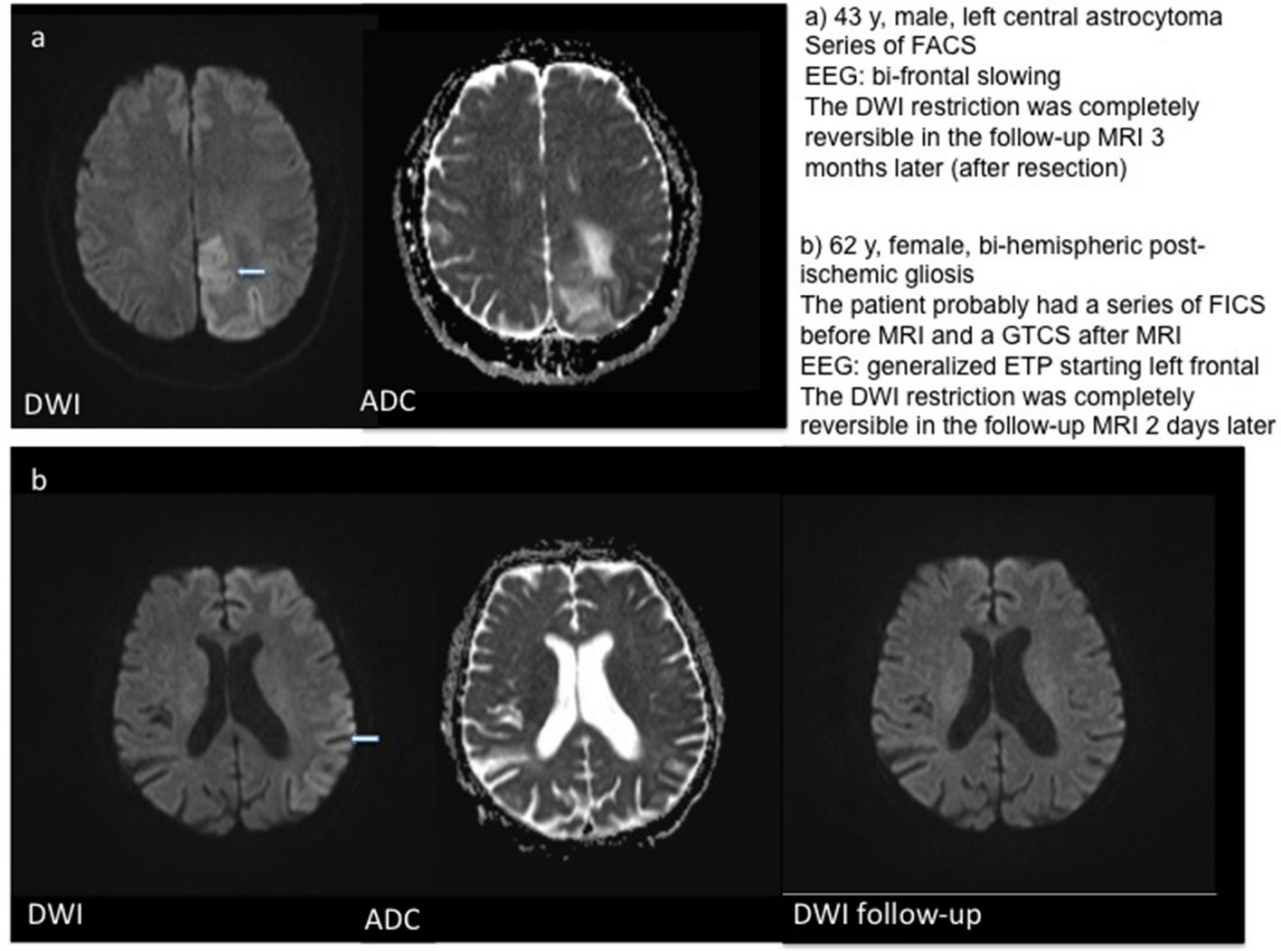
Examples for DWI restrictions in two patients with series of focal seizures. T2w–T2 weighted imaging.

**Figure 2:**
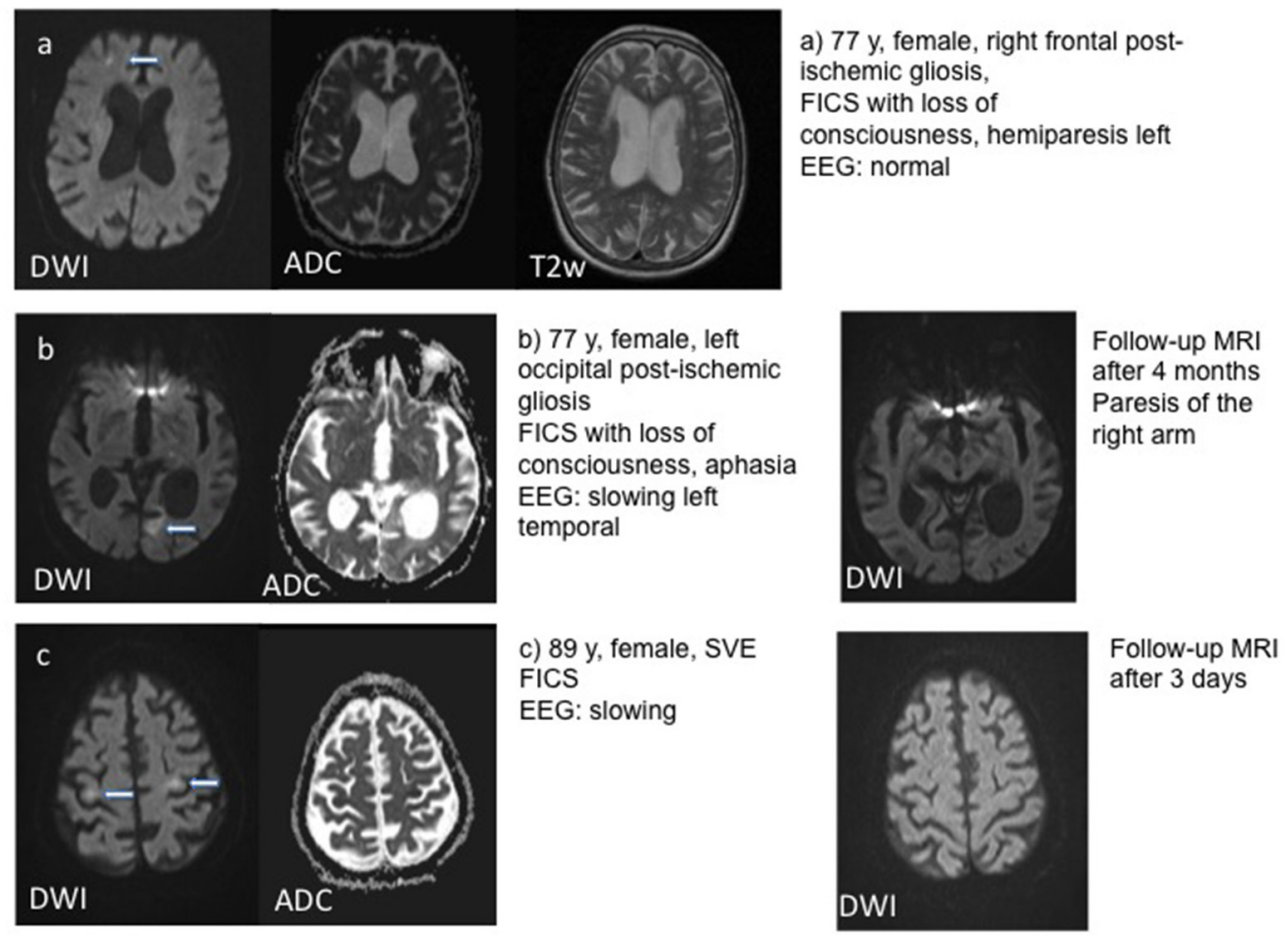
Examples for DWI restrictions in three patients with single focal seizures including follow-up MRI in patients **(b)** and **(c)**. T2w – T2 weighted imaging.

In three patients, a localized post-ischemic gliosis was identified as the presumed cause of epileptic activity. In these patients, DWI restriction was observed in the same area as the structural pathology. The same was the case for two patients with brain tumors and one patient with limbic encephalitis, who showed DWI alteration in the left temporal lobe and hippocampus. One patient showed post-ischemic gliosis in both hemispheres, but DWI restriction was localized on the left hemisphere (temporal hippocampal, parietal and thalamic) only. In patients with generalized bi-hemispheric pathology, like subcortical vascular encephalopathy (SVE) or cerebral amyloid angiopathy (CAA), DWI restriction was usually localized in one hemisphere in a single subject, but varied in-between subjects without preponderance of the left or right hemisphere. One patient with bilateral cysticercosis showed DWI changes only in the right thalamus. Patients with SE or seizure series mostly showed a typical “garland-like” pattern alongside the cortical band or on the border of a defined lesion, sometimes also thalamic (Figure 1). By contrast, patients with single focal seizures showed rather subtle and smaller DWI restriction patterns (Figure 2).

## Discussion

In this large retrospective study, we systematically analyzed DWI alterations related to epileptic seizures in a cohort of 454 patients who presented at a single center with epileptic seizures or SE and received MRI scanning within the first 24 h after seizure onset. DWI restrictions were observed in 4% of patients. In previous studies, changes of cerebral diffusivity as detected by MRI have been reported in epilepsy patients (11–13, 20, 21). Yet, DWI changes in epilepsy were described to be almost exclusively related to focal or generalized SE or series of seizures. However, in our unselected patient collective, we found DWI alterations not only in 56% of patients with focal SE or series of focal seizures and in 19% of all patients with SE, but also in 3% of patients after single focal seizures and 2.5% of patients after single GTCS. While in the case of SE patients, this percentage is in accordance with two large previous series, where DWI alterations were observed in 28 and 67% of patients (12, 13), MRI alterations in patients with a single focal or generalized seizure seem to be more common than assumed until now.

Several different pathomechanisms have been discussed to explain changes in brain diffusivity related to epileptic activity: one possible reason could be an energy deficit of the Na^+^/K^+^-ATPase pump after and during seizures, resulting in increased net uptake of sodium from extracellular to intracellular compartments, which is then followed by water influx into the cell, leading to cell swelling and cytotoxic edema (22). This in turn leads to decreased ADC and DWI restriction in the region of the epileptogenic focus. A failure of the Na^+^/K^+^-ATPase pump has in a similar way been shown in ischemic stroke (23). One other possible mechanism leading to focal cell damage may be an increase of calcium influx resulting in toxic calcium levels. This influx is due to an excessive release of excitatory neurotransmitters such as glutamate (24) during ictal activity. However, there might be a common mechanism shared with ischemic pathophysiology, i.e., terminal depolarization with the difference being that it is not terminal in ictal etiology. Furthermore, clinical experience as well as studies [e.g. (25)] indicate that DWI restrictions are less pronounced under ictal than under ischemic conditions. In this regard, one limitation of our study is the fact that we did not measure the amount of ADC reduction in quantitative values. In the chronic phase (1–14 days after seizure activity) gliosis and neural cell death could lead to increased interstitial water resulting in ADC-/DWI-alterations (26). In all cases, cellular alterations leading to imaging changes remain mainly focal, and thus, DWI alterations have even been proposed as a tool to localize the center of ictal activity (20, 21, 27). In previous studies, DWI restrictions were often observed in the temporal lobe (12), especially in the hippocampus (11). Some patients, however, showed DWI alterations in the thalamus (12, 13). In our group, we saw a widespread pattern of cerebral diffusivity changes with no clear preponderance of e.g., the temporal lobe or hippocampal structures. In addition, DWI restrictions after single seizures did not show the classical “garland-like” pattern alongside the cortical band, as it has been described before in patients with SE, but were usually much smaller and often more difficult to detect.

The observed frequency of approximately 3% postictal DWI findings after single seizures gives rise to the advice that other relevant etiologies have to be ruled out before marking them as postictal in order not to delay specific therapies of other etiologies, especially ischemic stroke.

In over 90% of patients who showed a DWI restriction, a structural brain pathology was identified as the cause of the ictal activity. This percentage was significantly higher than in the group of patients without DWI abnormalities. The lateralization pattern of DWI restriction correlated well with the pattern of structural cerebral pathology. This finding has been described before in patients with SE (8, 11, 21). The fact that these structural lesions are not necessarily located in the hippocampus or temporal lobe only could explain the fact that we saw a widespread pattern of DWI changes in our patient group, affecting cortical regions of all lobes in a similar proportion.

In conclusion, our study shows that a phenomenon which has been well-known in patients with prolonged seizure activity can also be observed after single seizures, and should not be mistaken for cerebral stroke, as it does not show the same temporal and spatial characteristics.

## Author contributions

AH, MS, and EP reviewed MRI data. KT collected clinical data. AH wrote the manuscript. MS, JK, SF, EP, and AL reviewed the manuscript.

### Conflict of interest statement

The authors declare that the research was conducted in the absence of any commercial or financial relationships that could be construed as a potential conflict of interest.
